# A Pan-Cancer Analysis Reveals the Prognostic and Immunotherapeutic Value of ALKBH7

**DOI:** 10.3389/fgene.2022.822261

**Published:** 2022-02-11

**Authors:** Kaijie Chen, Dongjie Shen, Lin Tan, Donglin Lai, Yuru Han, Yonggang Gu, Changlian Lu, Xuefeng Gu

**Affiliations:** ^1^ Shanghai Key Laboratory of Molecular Imaging, Zhoupu Hospital, Shanghai University of Medicine and Health Sciences, Shanghai, China; ^2^ School of Health Science and Engineering, University of Shanghai for Science and Technology, Shanghai, China; ^3^ Department of General Surgery, Ruijin Hospital Lu Wan Branch, Shanghai Jiaotong University School of Medicine, Shanghai, China; ^4^ Xiangya School of Medicine, The Affiliated Zhuzhou Hospital Xiangya Medical College CSU, Central South University, Changsha, China; ^5^ Department of TCM, Shanghai Pudong Hospital, Shanghai, China; ^6^ School of Pharmacy, Shanghai University of Medicine and Health Sciences, Shanghai, China

**Keywords:** ALKBH7, pan-cancer, prognosis, immunotherapy, immune infiltration

## Abstract

Recent studies have identified a role for ALKBH7 in the occurrence and progression of cancer, and this protein is related to cellular immunity and immune cell infiltration. However, the prognostic and immunotherapeutic value of ALKBH7 in different cancers have not been explored. In this study, we observed high ALKBH7 expression in 17 cancers and low expression in 5 cancers compared to paired normal tissues. Although ALKBH7 expression did not correlate relatively significantly with the clinical parameters of age (6/33), sex (3/33) and stage (3/27) in the cancers studied, the results of the survival analysis reflect the pan-cancer prognostic value of ALKBH7. In addition, ALKBH7 expression was significantly correlated with the TMB (7/33), MSI (13/33), mDNAsi (12/33) and mRNAsi (13/33) in human cancers. Moreover, ALKBH7 expression was associated and predominantly negatively correlated with the expression of immune checkpoint (ICP) genes in many cancers. Furthermore, ALKBH7 correlated with infiltrating immune cells and ESTIMATE scores, especially in PAAD, PRAD and THCA. Finally, the ALKBH7 gene coexpression network is involved in the regulation of cellular immune, oxidative, phosphorylation, and metabolic pathways. In conclusion, ALKBH7 may serve as a potential prognostic pan-cancer biomarker and is involved in the immune response. Our pan-cancer analysis provides insight into the role of ALKBH7 in different cancers.

## Introduction

The AlkB family consists of Fe (II) and α-ketoglutarate-dependent dioxygenases. Nine AlkB homologues have been identified, including ALKBH1-8 and FTO. Previous experimental studies have found that these proteins are involved in biological processes such as RNA modification and fatty acid metabolism and the DNA damage response (Wu et al., 2016; Bian et al., 2019; Rajecka et al., 2019). In addition, recent studies have also discovered the potential of the AlkB family to participate in immune responses. ALKBH5 regulates the immune response by controlling CD4^+^ T cells (Zhou et al., 2021), regulating lactic acid levels (Li et al., 2020), and regulating HMGB1 expression (Chen et al., 2021). Moreover, many studies have reported a role for the AlkB family in the development of BLCA, HNSC, LUAD and OV, and this family is involved in regulating the immune response (Fujii et al., 2013; Pilžys et al., 2019; Cai et al., 2021; Wu et al., 2021), suggesting that AlkB homologues may be promising therapeutic targets.

ALKBH7 is a member of the AlkB family. Multiple studies have shown that ALKBH7 participates in biological processes such as lipid metabolism and programmed necrosis (Wang et al., 2014). ALKBH7 deficiency increases body weight and body fat in mice (Solberg et al., 2013) and protects mouse hearts from ischaemia-reperfusion (IR) injury (Kulkarni et al., 2020). ALKBH7 plays a key role in the process of alkylation and oxidation-induced programmed necrosis (Fu et al., 2013) and drives tissue- and sex-specific necrotic cell death responses (Jordan et al., 2017). In addition, recent studies have identified a role for ALKBH7 in the progression of several cancers and its relationship with immune cell infiltration. ALKBH7 expression is significantly elevated in hepatocellular carcinoma and negatively correlates with CD4^+^ cells, macrophages and neutrophils (Peng et al., 2021). ALKBH7 is associated with overall survival in individuals with lung adenocarcinoma and negatively correlates with CD8^+^ T cells and macrophages (Wu et al., 2021). ALKBH7 correlates with the pathological stage of ovarian serous carcinoma and positively correlates with the infiltration of CD8^+^ T cells, dendritic cells and neutrophils (Cai et al., 2021). ALKBH7 is involved in cellular immunity and the proliferation of HeLa cervical cancer cell lines (Meng et al., 2019).

However, the prognostic value and immunological role of ALKBH7 in cancer have not been systematically investigated. In the present study, we explored changes in the expression and prognostic value of ALKBH7 in 33 cancers. Then, we investigated ALKBH7 expression in different cancer immune and molecular subtypes. In addition, we performed an in-depth study of the immune mechanism of ALKBH7 in different cancers to explore its potential immunotherapeutic value. Overall, this work provides evidence to elucidate the immunotherapeutic role of ALKBH7 in cancer, which may be helpful for further functional experiments.

## Materials and Methods

### Data Acquisition and Software Availability

The genomic and clinicopathological information, somatic mutation and stemness score data of 33 cancers were obtained from TCGA (https://cancergenome.nih.gov/) and UCSC Xena (https://xena.ucsc.edu/) database (Goldman et al., 2020). In addition, to obtain more normal tissue genomic data, we downloaded tumor and normal tissue gene expression data combined with TCGA and GTEx database on the UCSCXenaShiny (https://shiny.hiplot.com.cn/ucsc-xena-shiny/) website (Wang S. et al., 2021). R 4.1.0 was used to integrate, analyse the original data and visualize the results.

### Differential ALKBH7 Expression Analysis in the Normal, Tumor, Various Age, Gender, and Pathological Stage Tissues

The discrepancy of the gene expression between various types of cancer and paired normal tissues was investigated to explore whether ALKBH7 is associated with cancer development. Differential expression analysis of ALKBH7 has been investigated in a variety of cancers with patients’ age, gender and pathological stage by using wilcox test.

### Immunohistochemical Staining

The Human Protein Atlas (https://www.proteinatlas.org/) contains over 25,000 antibodies and a collection of over 10 million immunohistochemical (IHC) images (Thul and Lindskog, 2018). To further compare the expression of ALKBH7 gene in tumors and corresponding normal tissues, antibody-based ALKBH7 protein profiles using immunohistochemistry were obtained from the HPA database.

### Prognostic Analysis of ALKBH7 in Human Cancers

Univariate Cox regression analysis and Kaplan-Meier curve were used to analyse the relationship between ALKBH7 expression and clinical survival data including overall survival (OS), disease-specific survival (DSS), disease-free interval (DFI) and progression-free interval (PFI) in 33 cancers.

### Analysis of ALKBH7 Expression in Different Subtypes of Human Cancers

The TISIDB database (http://cis.hku.hk/TISIDB/) is an online integrated repository portal integrating multiple types of data resources in oncoimmunology (Ru et al., 2019). The relationship between ALKBH7 expression and immune or molecular subtypes of different cancer types was explored through the TISIDB database.

### Correlation Analysis of ALKBH7 Expression With Immune Checkpoint Genes, Tumor Mutational Burden, Microsatellite Instability and Tumor Stemness Index in Human Cancers

The correlation between ALKBH7 expression and the expression of immune checkpoint (ICP) genes, was explored *via* the SangerBox website (http://sangerbox.com/). The tumor mutational burden (TMB), microsatellite instability (MSI) score and tumor stemness index of each TCGA tumor case were obtained from somatic mutation data and previously published studies respectively (Topalian et al., 2015; Bonneville et al., 2017). Tumor stemness indices are indicators for assessing the degree of oncogenic dedifferentiation. Among them, mRNAsi is a gene expression-based stemness index while mDNAsi is a DNA methylation-based stemness index. Correlations between ALKBH7 expression and TMB, MSI, mRNAsi and mDNAsi were analyzed using Spearman’s method.

### Analysis of Immune Infiltration-Related Factors and Pathways

The TIMER database (https://cistrome.shinyapps.io/timer/), which collected 10,897 samples across 32 cancer types from TCGA, was created to analyze the level of tumor-associated immune cell infiltration in the TME (Li et al., 2016; Li et al., 2017). The correlation between ALKBH7 expression and six immune cells (B cells, CD4^+^ T cells, CD8^+^ T cells, neutrophils, macrophages and dendritic cells) and tumor-infiltrating lymphocyte (TIL) marker genes in human cancers was investigated using the TIMER database. ESTIMATE is an algorithm that predicts the presence of immune and stromal cells in tumor tissue which based on gene expression profiles (Yoshihara et al., 2013). We calculated the stromal score, immune score and estimate socre of each case by using the ESTIMATE package. xCell is a powerful web tool for inferring the proportion of immune cell subtypes in tumor tissue (Aran et al., 2017). A spearman correlation heat map of ALKBH7 expression with 36 immunoinfiltrating subtypes of cells in human cancers was established. Finally, to further investigate the relevant signalling pathways, gene set enrichment analysis (GSEA) was performed to explore pathways of ALKBH7 coexpression gene network.

## Results

### Clinical Landscape of ALKBH7 Expression in 33 Cancers

**FIGURE 1:**
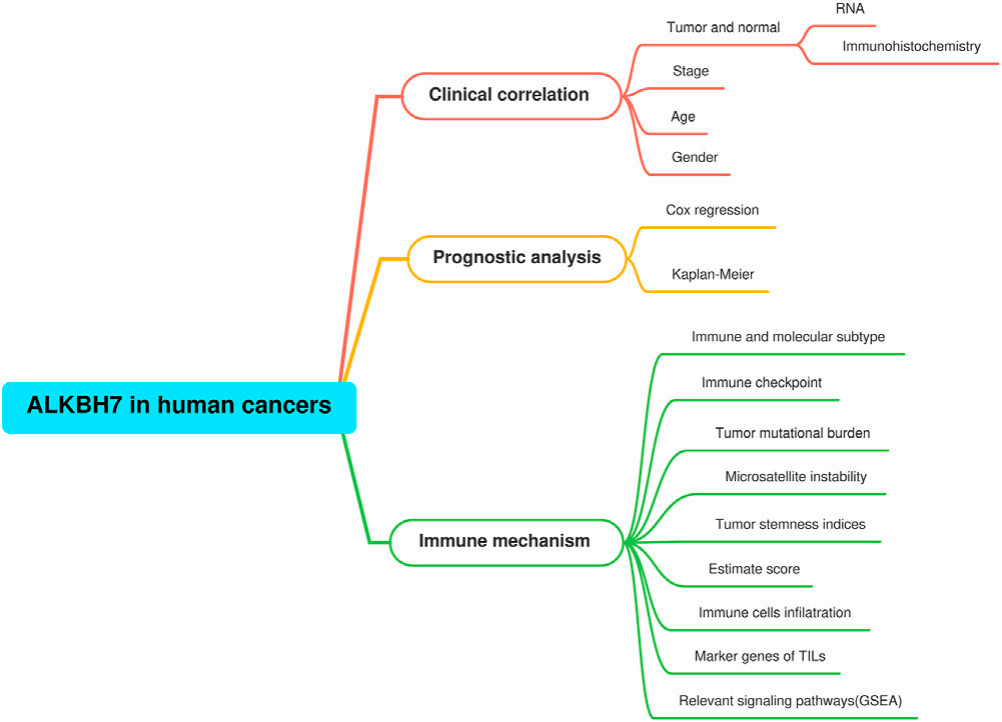
The analysis and indicators employed in our research. In clinical correlation section, differential ALKBH7 expression analyses were performed between different tissues (tumor versus normal), ages (≤60 *versus* >60), genders (male versus female), stages (stage I + II versus stage III + IV). Prognostic analysis was based on univariate Cox regression and Kaplan-Meier survival curve. In immune mechanism section, relevant signaling pathways were explored by GSEA based on the ALKBH7 expression.

**FIGURE 2:**
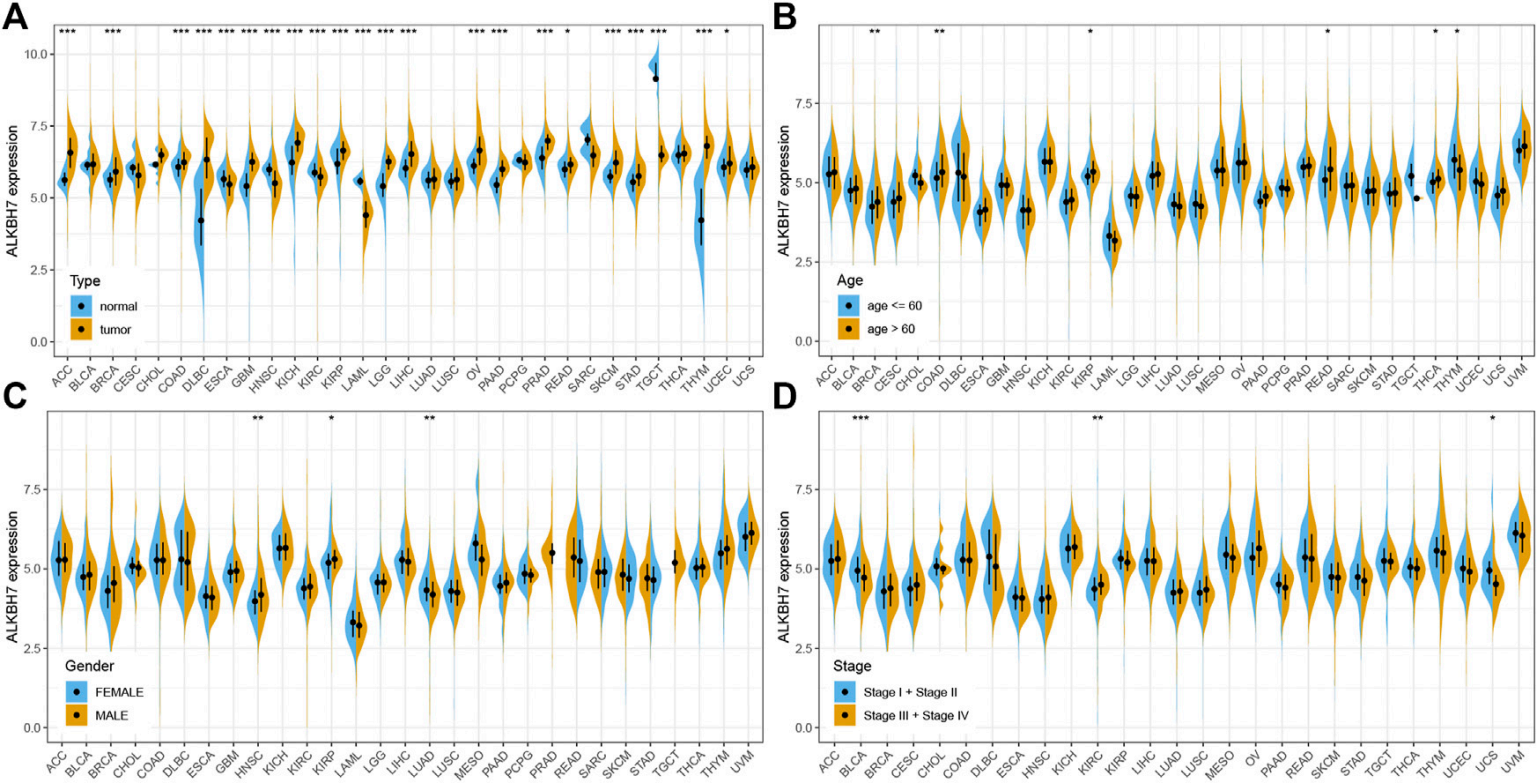
The clinical correlation of ALKBH7 expression. **(A)** Differential expression of ALKBH7 in normal and tumor samples from patients with 33 cancers; the correlations of ALKBH7 with age **(B)**, sex **(C)** and stage **(D)** in 33 cancers. “*” indicates *p* < 0.05, “**” indicates *p* < 0.01 and “***” indicates *p* < 0.001.

**FIGURE 3:**
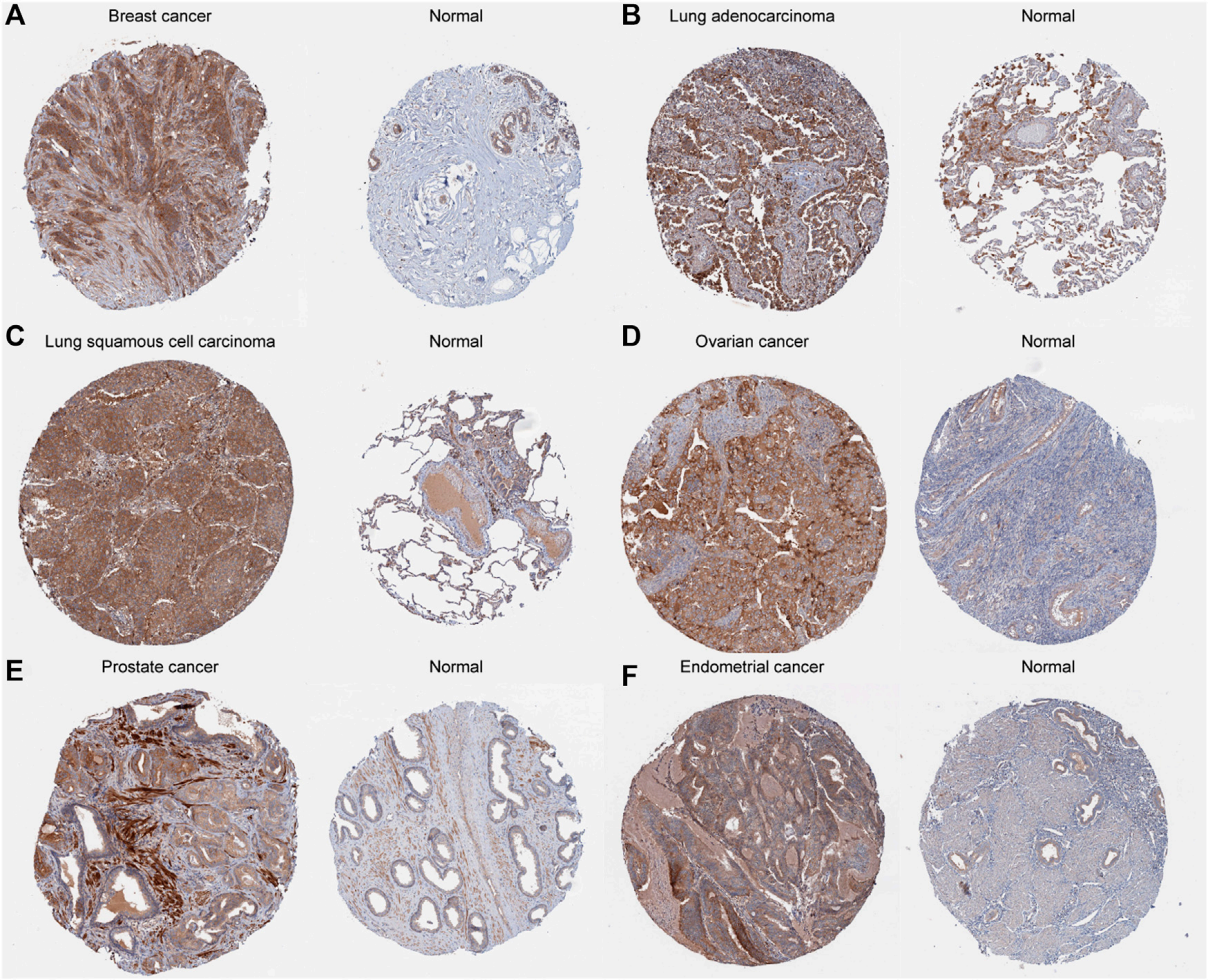
Representative ALKBH7 immunohistochemical staining in tumor and normal tissues. The expression of ALKBH7 gene in BRCA **(A)**, LUAD **(B)**, LUSC **(C)**, OV **(D)**, PRAD **(E)**, and UCEC **(F)** is significantly higher than that in the corresponding normal tissues.

The details of the analysis are summarized and presented in Figure 1 for a more comprehensive perspective. As illustrated in Figure 2A, significantly higher ALKBH7 expression was detected in most human cancers than in adjacent normal tissues, such as ACC, BRCA, COAD, DLBC, GBM, KICH, KIRP, LGG, LIHC, OV, PAAD, PRAD, READ, SKCM, STAD, THYM and UCEC. In contrast, significantly lower ALKBH7 expression was observed in a few human cancers (ESCA, HNSC, KIRC, LAML and TGCT). ALKBH7 was highly differentially expressed among elderly patients in the THCA, BRCA, KIRP, READ and COAD groups, whereas it was weakly expressed in patients with THYM (Figure 2B). Meanwhile, the results indicated significant sex-based differences in ALKBH7 expression in HNSC, KIRP and LUAD (Figure 2C). In addition, ALKBH7 expression was significantly correlated with the pathological stage of some cancers, including BLCA, KIRC and UCS (Figure 2D). Finally, we used immunohistochemistry to validate ALKBH7 expression. Compared with normal tissues, ALKBH7 was highly expressed in BRCA, LUAD, LUSC, OV, PRAD and UCEC (Figure 3).

### Pan-Cancer Analysis of the Multifaceted Prognostic Value of ALKBH7

**FIGURE 4:**
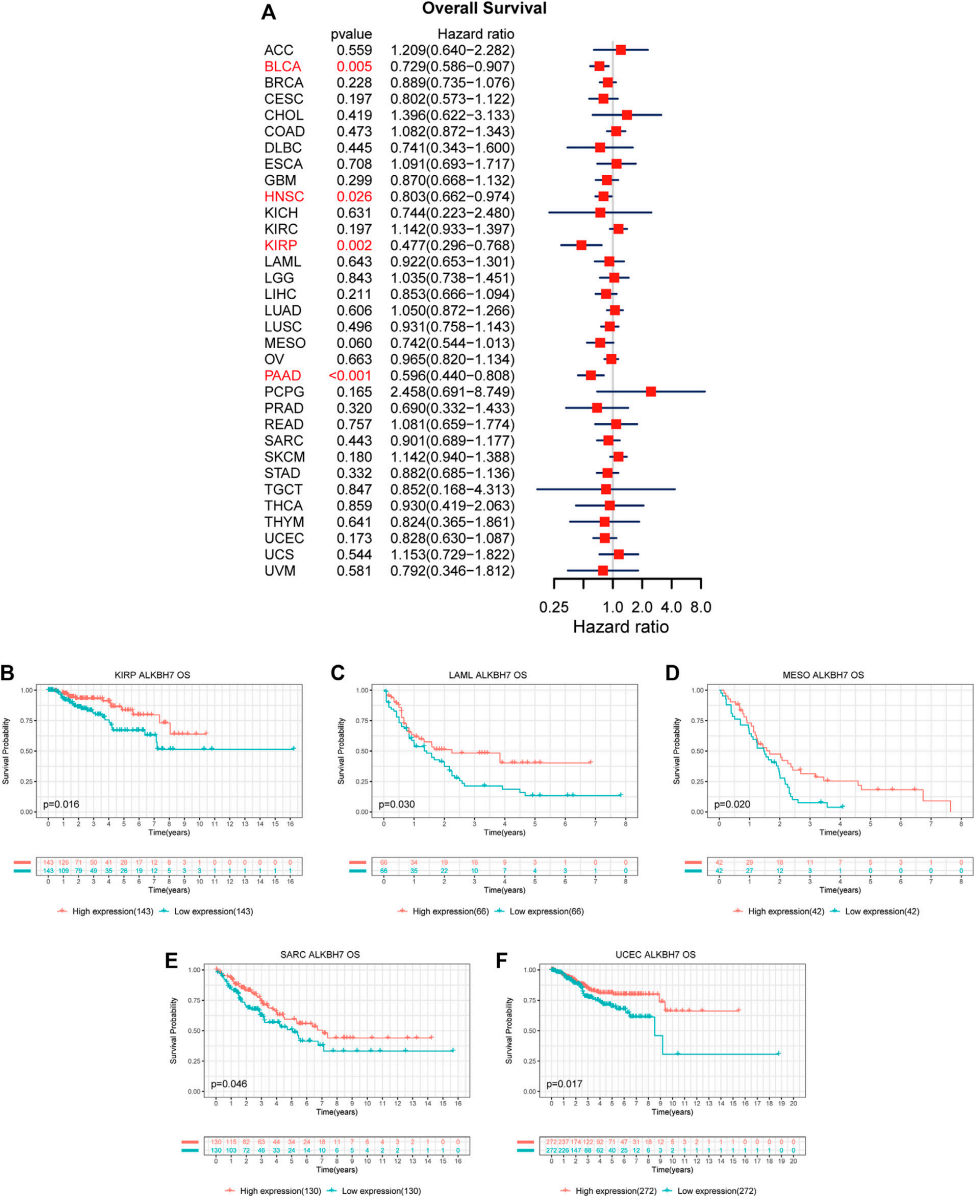
Associations between ALKBH7 expression and OS of patients with cancer. **(A)** Forest plot showing the hazard ratios of ALKBH7 in 33 cancers; Kaplan-Meier survival curves of OS forpatients stratified according to different ALKBH7 expression profiles in KIRP **(B)**, LAML **(C)**, MESO **(D)**, SARC **(E)** and UCEC **(F)**.

**FIGURE 5:**
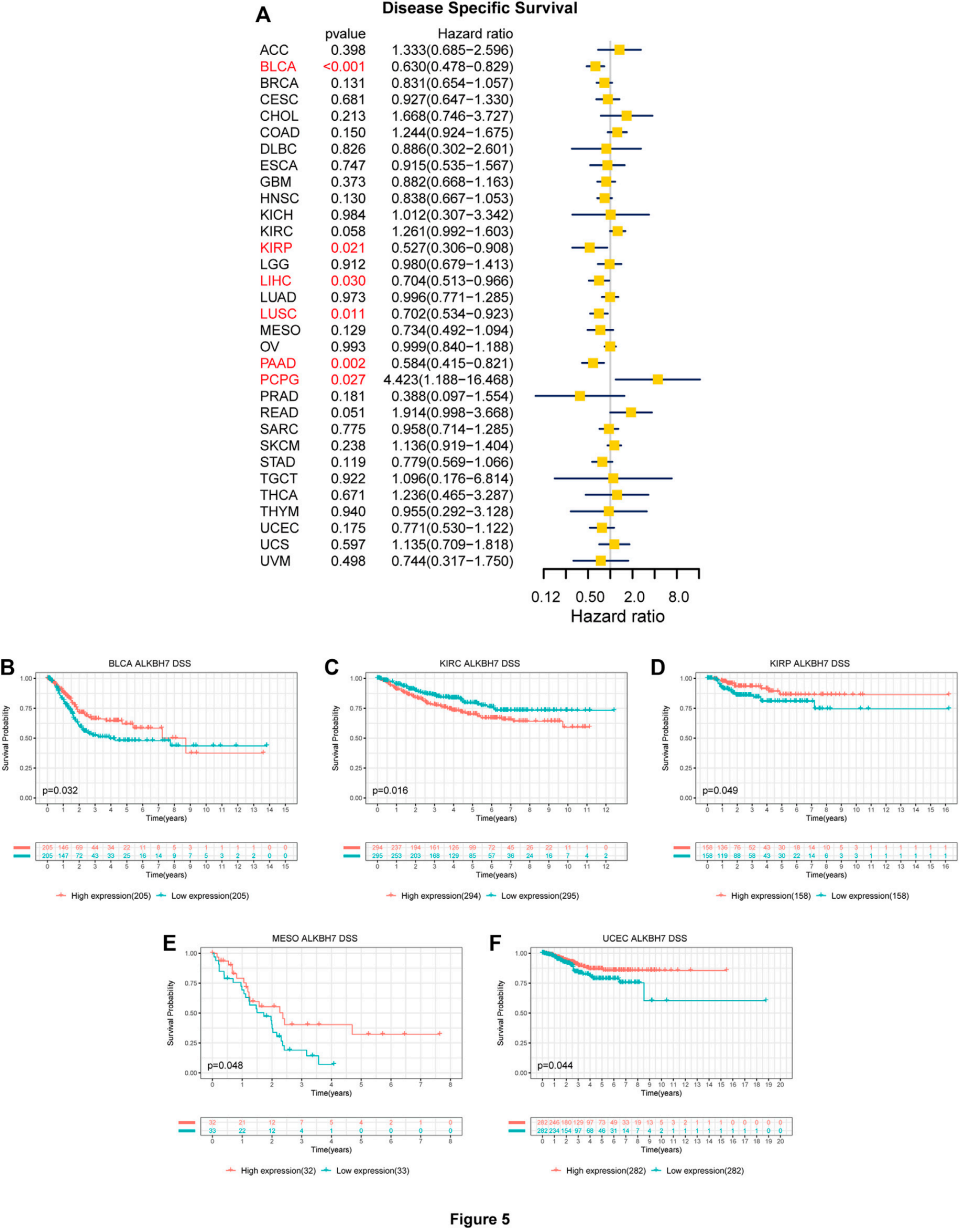
Associations between ALKBH7 expression and DSS of patients with cancer. **(A)** Forest plot showing hazard ratios of ALKBH7 in 32 cancers; Kaplan-Meier survival curves of DSS forpatients stratified according to different ALKBH7 expression profiles in BLCA **(B)**, KIRC **(C)**, KIRP **(D)**, MESO **(E)** and UCEC **(F)**.

The association between ALKBH7 expression and patient prognosis was estimated in the pan-cancer dataset. The survival metrics included OS, DSS, DFI, and PFI. Univariate Cox regression analysis of the results from 33 types of cancer suggested that ALKBH7 expression significantly correlated with OS in 4 types of cancer, including BLCA, HNSC, KIRP, and PAAD. Kaplan–Meier survival curves indicated that downregulated ALKBH7 expression was remarkably associated with shorter OS of patients with KIRP, LAML, MESO, SARC, and UCEC (Figure 4). The relationship between ALKBH7 expression and DSS in patients with cancer was examined. ALKBH7 expression affected DSS in six types of cancer, including BLCA, KIRP, LIHC, LUSC, PAAD, and PCPG. The Kaplan–Meier analysis indicated that decreased ALKBH7 expression indicated shorter DSS of patients with BLCA, KIRP, MESO, and UCEC, while increased ALKBH7 expression corresponded with shorter DSS of patients with KIRC (Figure 5). Cox regression analysis of the DFI revealed that ALKBH7 expression significantly correlated with DFI in 4 types of cancer, including LUSC, OV, PAAD, and THCA. The results from the Kaplan–Meier analysis suggested that increased ALKBH7 expression was associated with a poor prognosis for patients with PRAD, while decreased ALKBH7 expression was associated with a poor prognosis for patients with THCA (Supplementary Figure S1). We also assessed the association between ALKBH7 expression and PFI and identified that ALKBH7 expression influenced PFI in patients with BLCA, KIRC, LUSC and PAAD. Kaplan–Meier PFI curves revealed that decreased ALKBH7 mRNA expression correlated with an unfavourable PFI in patients with BLCA and PAAD. In contrast, increased ALKBH7 mRNA expression correlated with an unfavourable PFI in patients with KIRC (Supplementary Figure S2).

### ALKBH7 Expression Is Related to Immune and Molecular Subtypes in Human Cancers

**FIGURE 6:**
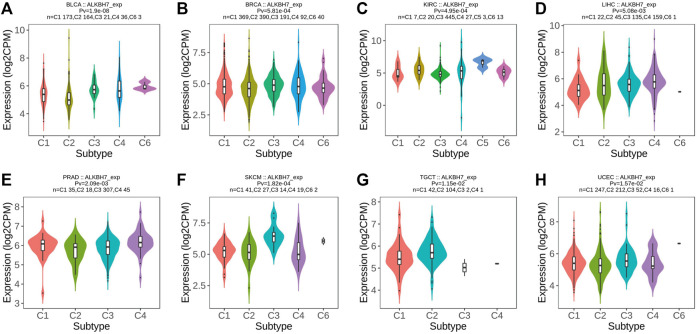
The relationship between ALKBH7 expression and immune subtypes in BLCA **(A)**, BRCA **(B)**, KIRC **(C)**, LIHC **(D)**, PRAD **(E)**, SKCM **(F)**, TGCT **(G)** and UCEC **(H)**. [C1 (wound healing); C2 (IFN-gamma dominant); C3 (inflammatory); C4 (lymphocyte depleted); C5 (immunologically quiet); C6 (TGF-beta dominant)].

**FIGURE 7:**
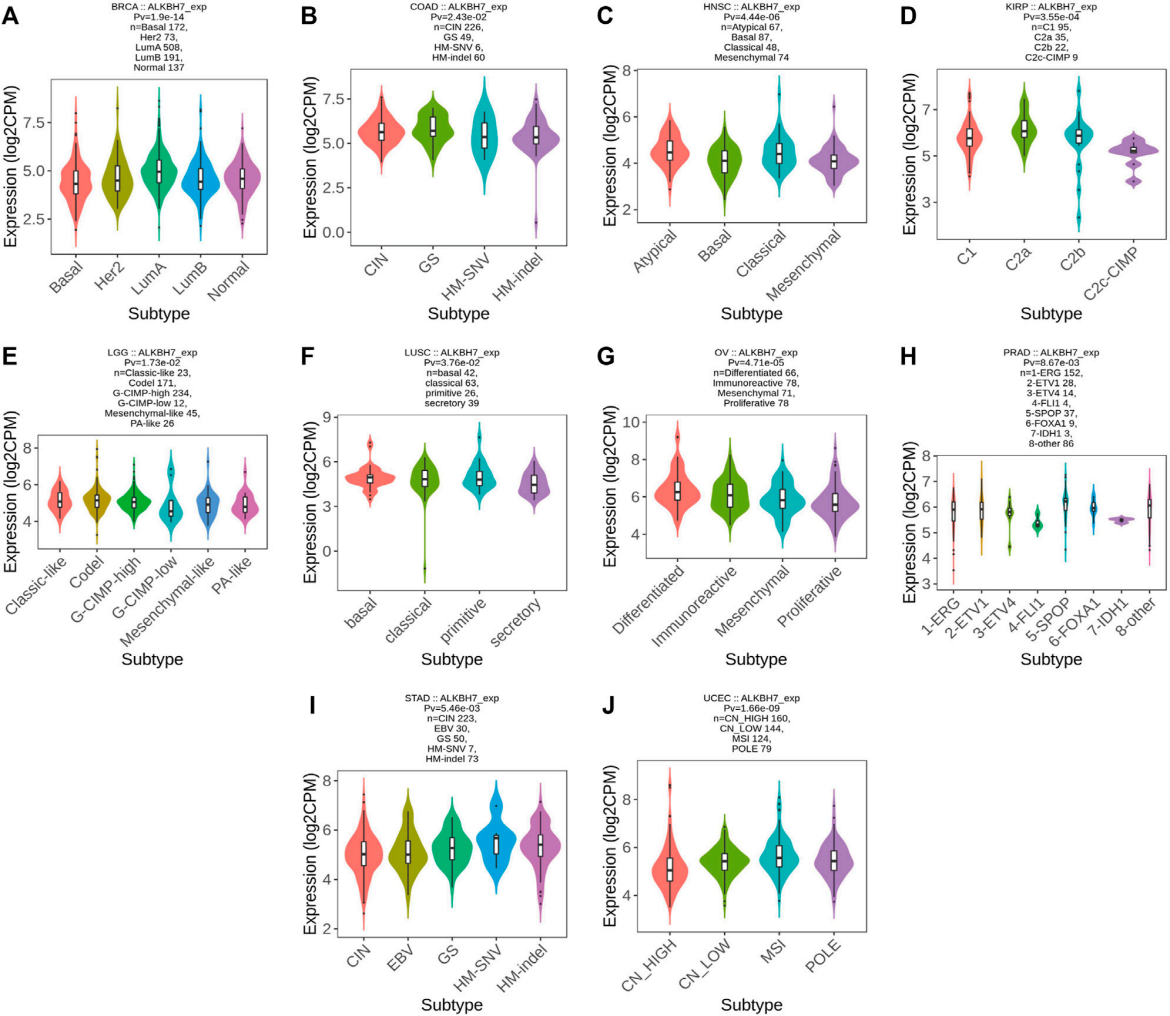
The relationship between ALKBH7 expression and molecular subtypes in BRCA **(A)**, COAD **(B)**, HNSC **(C)**, KIRP **(D)**, LGG **(E)**, LUSC **(F)**, OV **(G)**, PRAD **(H)**, STAD **(I)** and UCEC **(J)**.

Based on accumulating evidence, immunophenotyping reflects the comprehensive immune status of a tumor, which is closely related to immunotherapy and the tumor microenvironment (Ma et al., 2021). Different molecular subtypes correspond to the unique molecular biology of cancer and may facilitate the selection of molecular targeted therapies and immunotherapy strategies (Kim et al., 2019; Bai et al., 2021). Next, ALKBH7 expression in immune and molecular subtypes of human cancer was explored using the TISIDB website. ALKBH7 expression was significantly different in different immune subtypes of BLCA, BRCA, KIRC, LIHC, PRAD, SKCM, TGCT, and UCEC (Figure 6). In addition, the trends for the up- and downregulation of ALKBH7 expression were also different in different immune subtypes of a specific cancer type. Taking SKCM as an example, low ALKBH7 expression was detected in C2 and C4 types and high expression was observed in the C3 type. Regarding different molecular subtypes of cancers, a significant correlation with ALKBH7 expression was observed in BRCA, COAD, HNSC, KIRP, LGG, LUSC, OV, PRAD, STAD and UCEC (Figure 7). Based on the results described above, we suggest that ALKBH7 may play an important role in the tumor immune microenvironment and modulate the effect of immunotherapy.

### ALKBH7 Expression Is Related to Immune Checkpoint Genes in Human Cancers

**FIGURE 8:**
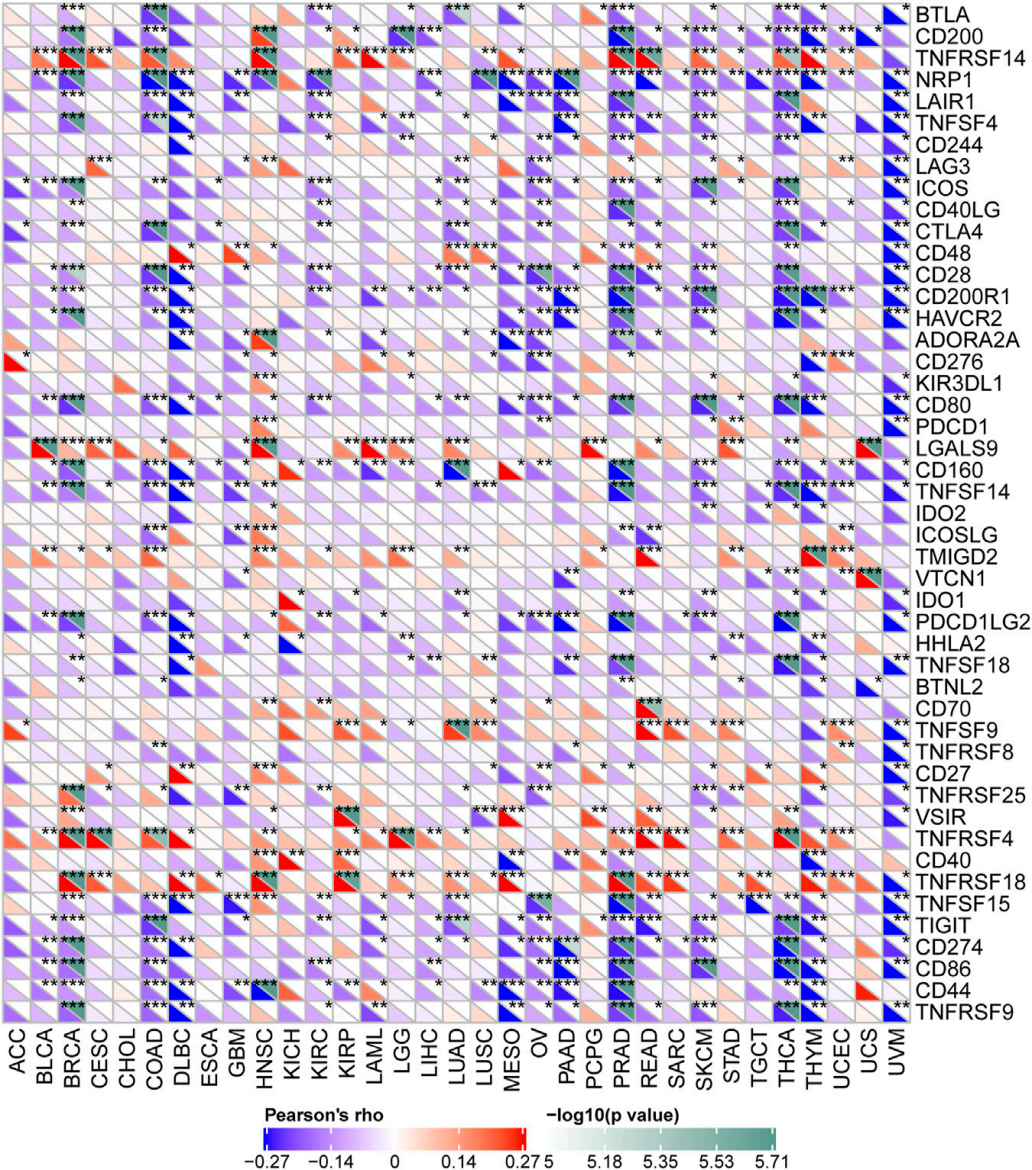
Correlations between the expression of ALKBH7 and immune checkpoint genes in 33 types of cancer. “*” indicates *p* < 0.05, “**” indicates *p* < 0.01 and “***” indicates *p* < 0.001.

Studies have shown that immune checkpoint genes have important implications for immunotherapy in many cancers (Topalian et al., 2015; Li et al., 2019). Here, we collected expression patterns of 47 common immune checkpoint genes and analysed the relationship between ALKBH7 expression and immune checkpoint gene expression to explore the potential role of ALKBH7 in immunotherapy. As shown in Figure 8, ALKBH7 expression significantly correlated with the expression of most ICP genes in many cancers, such as BRCA COAD, HNSC, KIRC, LUAD, OV, PAAD, PRAD, READ, SKCM, THCA, THYM, and UVM. Among them, a negative correlation was the main trend; for example, in PRAD, ALKBH7 expression was negatively correlated with the expression of 30 ICP genes and positively correlated with the expression of 5 ICP genes. Thus, high levels of ALKBH7 expression may predict unsatisfactory immunotherapy outcomes when targeting ICP genes. On the other hand, ALKBH7 inhibitors may be potential alternative therapeutic approaches. Therefore, we hypothesized that ALKBH7, a potential pan-cancer biomarker or a novel immunotherapeutic target, may predict the response to immunotherapy or achieve promising therapeutic outcomes, respectively.

### ALKBH7 Expression Is Related to the Tumor Mutational Burden, Microsatellite Instability, and Tumor Stemness Index

**FIGURE 9:**
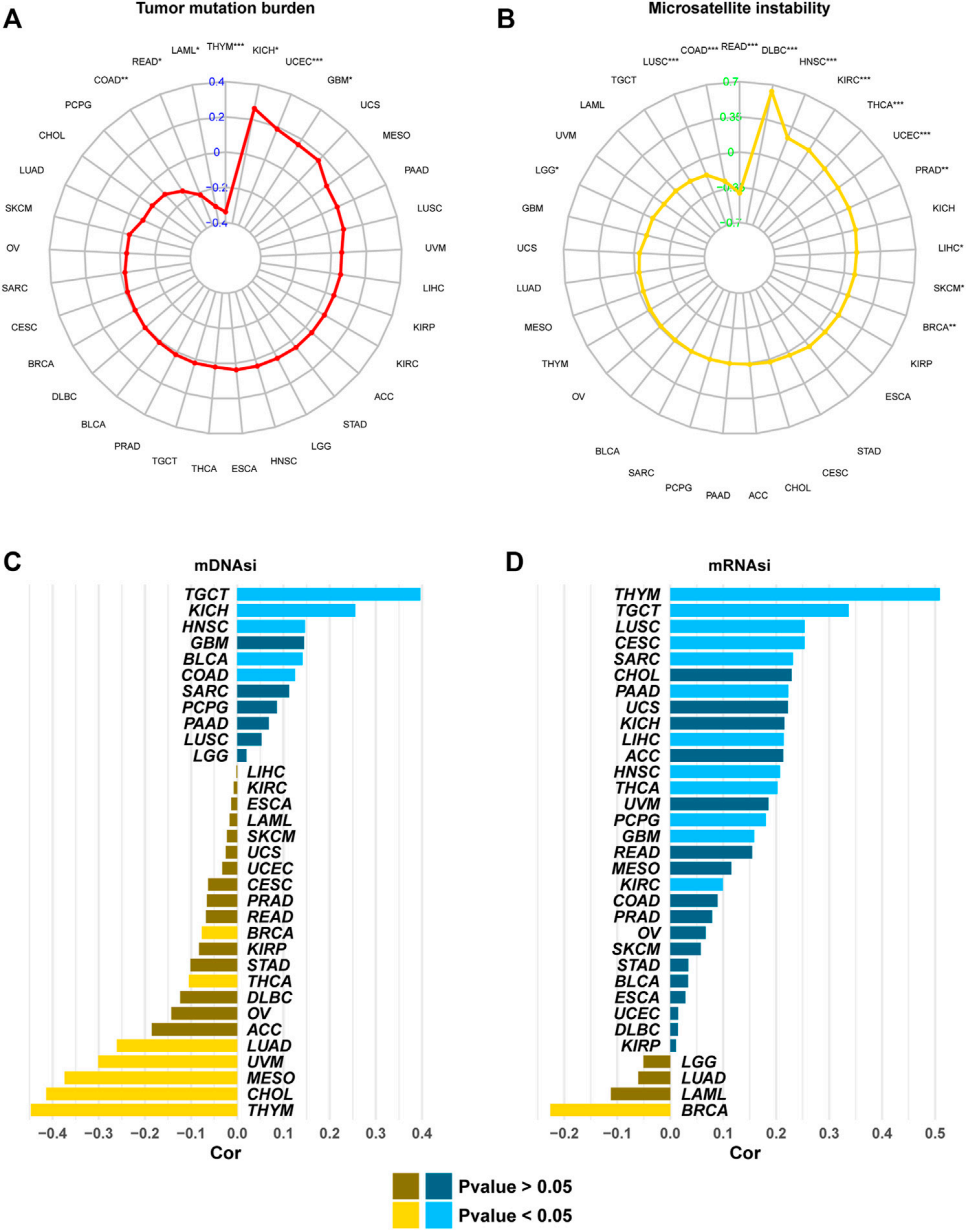
The correlation between ALKBH7 expression and the TMB **(A)**, MSI **(B)**, mDNAsi **(C)**, and mRNAsi **(D).** “*” indicates *p* < 0.05, “**” indicates *p* < 0.01 and “***” indicates *p* < 0.001.

We analysed the correlations between ALKBH7 expression and the TMB, MSI, and tumor stemness index to explore the role of ALKBH7 in the immune mechanism and immune response of the tumor microenvironment (TME). The TMB, MSI, and tumor stemness index in the tumor microenvironment are related to antitumor immunity and might predict the therapeutic efficacy of tumor immunotherapy (Lee et al., 2016; Yarchoan et al., 2017; Malta et al., 2018). As presented in Figure 9, ALKBH7 was associated with the TMB in 7 cancers and MSI in 13 cancers. In addition, ALKBH7 was related to mDNAsi in 12 cancers and mRNAsi in 13 cancers. Among them, ALKBH7 expression was negatively correlated with the TMB and MSI in COAD and READ, while it was positively correlated with the TMB and MSI in UCEC. Based on this finding, ALKBH7 might exert an indirect effect on the immunotherapeutic response of COAD, READ and UCEC. ALKBH7 was positively correlated with mRNAsi and mDNAsi in TGCT and HNSC, but negatively correlated with mRNAsi and mDNAsi in BRCA. High ALKBH7 expression in TGCT and HNSC may be related to the low sensitivity to immune checkpoint blockade therapy; in contrast, high ALKBH7 expression in BRCA may be related to the high sensitivity to immune checkpoint blockade therapy. Interestingly, ALKBH7 was positively correlated with mRNAsi but negatively correlated with mDNAsi in THCA and THYM. This result might arise from the discrepancies between mRNAsi and mDNAsi caused by DNA hypermethylation (Malta et al., 2018).

### Correlation Analysis Between ALKBH7 Expression and Infiltrating Immune Cells and the ESTIMATE Score

**FIGURE 10:**
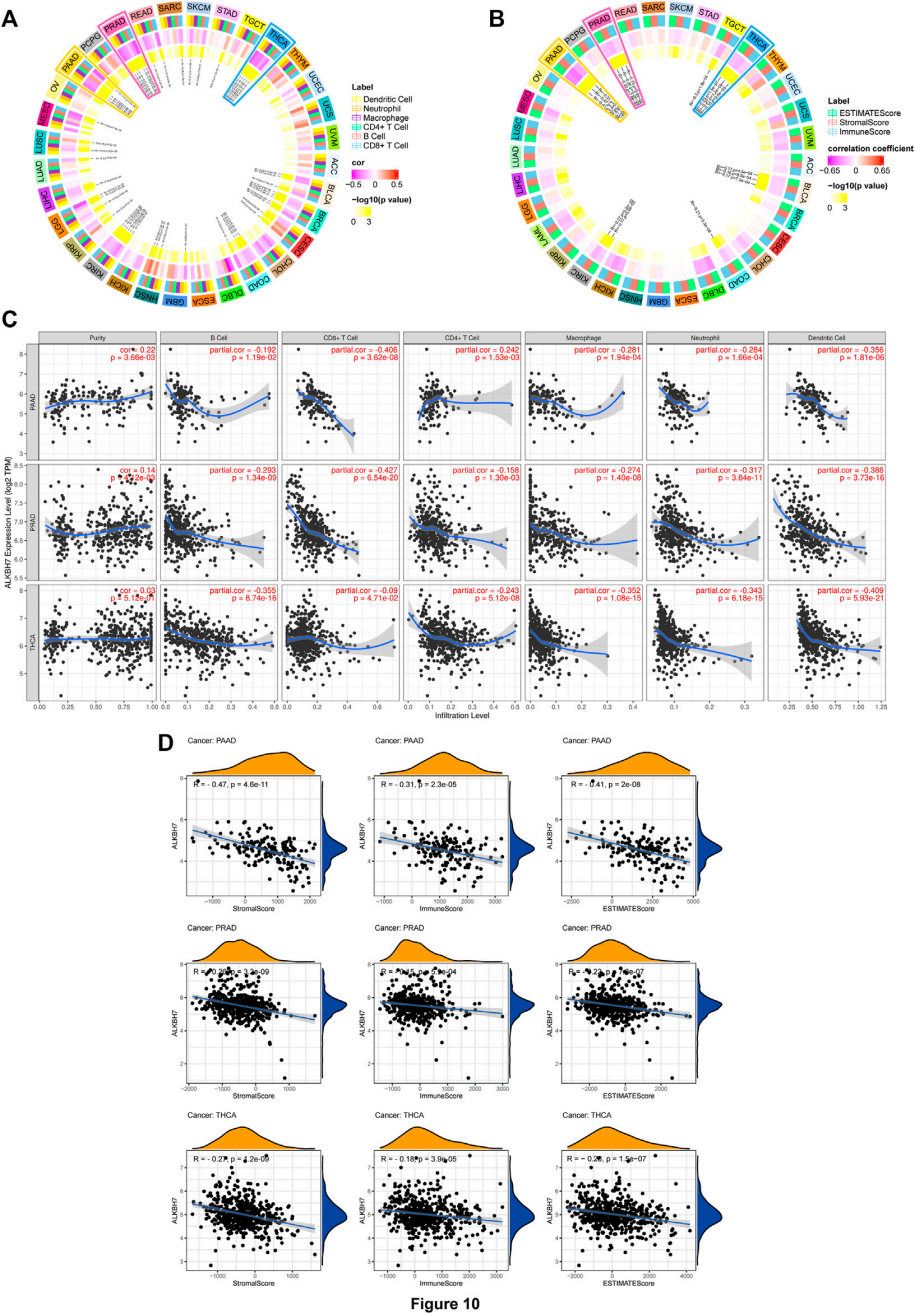
Correlations between ALKBH7 expression and both immune cell infiltration and ESTIMATE score. **(A)** The relationship between the ALKBH7 expression level and numbers of infiltrating B cells, CD4^+^ T cells, CD8^+^ T cells, macrophages, neutrophils, dendritic cell in human cancers. **(B)** The relationship between ALKBH7 expression and the ESTIMATE score in human cancers. **(C)** Correlation of ALKBH7 expression with immune cell infiltration levels in PAAD, PRAD, and THCA. **(D)** Correlation of ALKBH7 expression with ESTIMATE scores in PAAD, PRAD, and THCA.

The tumor microenvironment contains immune cells and fibroblasts, which affect the effect of immunotherapy (Aran et al., 2015). We analysed the correlation between ALKBH7 expression and six types of infiltrating immune cells, including B cells, CD4^+^ T cells, CD8^+^ T cells, neutrophils, macrophages, and dendritic cells. The results revealed a significant correlation in 31 cancer types. ALKBH7 expression displayed a strong relationship with dendritic cells in 8 cancer types, macrophages in 9 cancer types, neutrophils in 11 cancer types, CD8^+^ T cells in 14 cancer types, B cells in 8 cancer types and CD4^+^ T cells in 3 cancer types (Figure 10A). Results from the TIMER database included these results, and all details are shown in Supplementary Table S1. Subsequently, the correlation between ALKBH7 expression and stromal, immune and ESTIMATE scores was analysed (Figure 10B). All results are presented in Supplementary Table S2. Interestingly, the most significant correlation between ALKBH7 expression and the two parameters described above was observed in PAAD, PRAD and THCA. As shown in Figures 10C,D, ALKBH7 expression was negatively correlated with TILs and stromal, immune, and ESTIMATE scores. Therefore, ALKBH7 may be involved in inhibiting immune cell infiltration in PAAD, PRAD and THCA.

**FIGURE 11:**
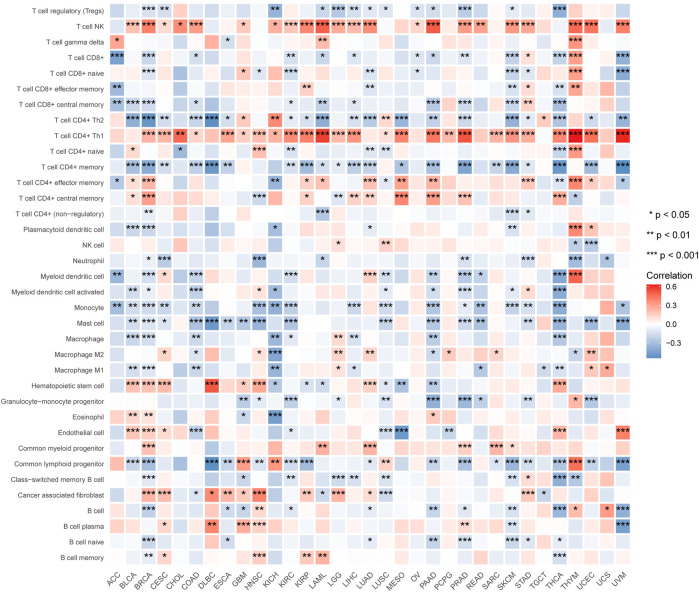
Heat map of the correlations between ALKBH7 expression and immune cell subtypes in 33 types of cancer. “*” indicates *p* < 0.05, “**” indicates *p* < 0.01 and “***” indicates *p* < 0.001.

We also used the xCell web tool to explore the association between ALKBH7 gene expression and the infiltration of various subtypes of immune cells. The Spearman correlation heat map is shown in Figure 11. NK T cells and CD4^+^ Th1 T cells were positively correlated with ALKBH7 gene expression in most cancers. In contrast, CD4^+^ Th2 T cells, memory CD4^+^ T cells, monocytes and mast cells were negatively correlated with ALKBH7 gene expression in most cancers. In PAAD, PRAD and THCA, ALKBH7 expression was associated with most subtypes of immune cells and generally exhibited negative correlations.

### Correlation Between ALKBH7 Expression and Various Immune Markers

We validated the correlations between ALKBH7 expression and diverse immune signatures in PAAD, PRAD and THCA using the TIMER database to obtain a better understanding of ALKBH7 crosstalk with the immune response. The genes listed in Table 1 were used to characterize immune cells, including CD8^+^ T cells, T cells, B cells, monocytes, tumor-associated macrophages (TAMs), M1 macrophages, M2 macrophages, neutrophils and dendritic cells. Tumor purity is an important aspect affecting the number of infiltrating immune cells in clinical cancer biopsies. After adjusting for tumor purity, ALKBH7 expression was significantly negatively correlated with most of the immune markers of divergent types of immune cells in PAAD, PRAD and THCA (Table 1).

**TABLE 1:** Correlation analysis between ALKBH7 and gene markers of immune cells in TIMER.

Description	Gene markers	THCA				PRAD				PAAD			
		None		Purity		None		Purity		None		Purity	
		Cor	Pvalue	Cor	Pvalue	Cor	Pvalue	Cor	Pvalue	Cor	Pvalue	Cor	Pvalue
CD8^+^ T cell	CD8A	−0.202	^a^	−0.192	^a^	−0.314	^a^	−0.254	^a^	−0.292	^a^	−0.233	^b^
	CD8B	−0.152	^a^	−0.143	^b^	−0.157	^a^	−0.124	^c^	−0.279	^a^	−0.217	^b^
T cell (general)	CD3D	−0.26	^a^	−0.25	^a^	−0.153	^a^	−0.071	0.146	−0.19	^c^	−0.122	0.112
	CD3E	−0.305	^a^	−0.295	^a^	−0.259	^a^	−0.2	^a^	−0.238	^b^	−0.17	^c^
	CD2	−0.316	^a^	−0.303	^a^	−0.253	^a^	−0.16	^b^	−0.292	^a^	−0.224	^b^
B cell	CD19	−0.183	^a^	−0.167	^a^	−0.059	0.187	−0.015	0.759	−0.186	^c^	−0.118	0.123
	CD79A	−0.209	^a^	−0.194	^a^	−0.153	^a^	−0.089	0.068	−0.194	^b^	−0.123	0.109
Monocyte	CD86	−0.404	^a^	−0.393	^a^	−0.414	^a^	−0.336	^a^	−0.399	^a^	−0.332	^a^
	CSF1R	−0.346	^a^	−0.343	^a^	−0.37	^a^	−0.299	^a^	−0.347	^a^	−0.289	^a^
TAM	CCL2	−0.26	^a^	−0.244	^a^	−0.223	^a^	−0.165	^a^	−0.282	^a^	−0.253	^a^
	CD68	−0.358	^a^	−0.34	^a^	−0.35	^a^	−0.289	^a^	−0.228	^b^	−0.157	^c^
	IL10	−0.292	^a^	−0.279	^a^	−0.338	^a^	−0.251	^a^	−0.258	^a^	−0.208	^b^
M1 Macrophage	IRF5	−0.324	^a^	−0.317	^a^	−0.234	^a^	−0.263	^a^	0.027	0.719	0.058	0.454
	PTGS2	−0.319	^a^	−0.31	^a^	−0.268	^a^	−0.179	^a^	−0.237	^b^	−0.253	^a^
M2 Macrophage	CD163	−0.335	^a^	−0.32	^a^	−0.414	^a^	−0.343	^a^	−0.438	^a^	−0.377	^a^
	VSIG4	−0.353	^a^	−0.345	^a^	−0.379	^a^	−0.303	^a^	−0.347	^a^	−0.278	^a^
	MS4A4A	−0.334	^a^	−0.321	^a^	−0.363	^a^	−0.297	^a^	−0.379	^a^	−0.309	^a^
Neutrophils	CEACAM8	−0.196	^a^	−0.197	^a^	0.004	0.922	0.017	0.724	−0.059	0.433	−0.005	0.95
	ITGAM	−0.411	^a^	−0.401	^a^	−0.361	^a^	−0.309	^a^	−0.285	^a^	−0.191	^c^
	CCR7	−0.293	^a^	−0.276	^a^	−0.274	^a^	−0.217	^a^	−0.178	^c^	−0.124	0.106
Dendritic cell	HLA-DPB1	−0.305	^a^	−0.295	^a^	−0.119	^b^	−0.057	0.246	−0.232	^b^	−0.159	^c^
	HLA-DQB1	−0.291	^a^	−0.295	^a^	−0.198	^a^	−0.147	^b^	−0.243	^b^	−0.189	^c^
	HLA-DRA	−0.377	^a^	−0.365	^a^	−0.351	^a^	−0.295	^a^	−0.35	^a^	−0.289	^a^
	HLA-DPA1	−0.364	^a^	−0.351	^a^	−0.339	^a^	−0.257	^a^	−0.356	^a^	−0.3	^a^
	CD1C	−0.336	^a^	−0.319	^a^	−0.319	^a^	−0.242	^a^	−0.199	^b^	−0.14	0.067
	NRP1	−0.258	^a^	−0.245	^a^	−0.29	^a^	−0.271	^a^	−0.471	^a^	−0.439	^a^
	ITGAX	−0.363	^a^	−0.348	^a^	−0.299	^a^	−0.277	^a^	−0.185	^c^	−0.091	0.236

c *p* < 0.05.

b *p* < 0.01.

a *p* < 0.001.

We also examined the correlations between ALKBH7 expression and various functional T cells, including Th1, Th1-like, Th2, Th17, Tfh, Treg, resting Tregs, effector Tregs, effector T cells, naïve T cells, effector memory T cells, resistant memory T cells, and exhausted T cells (Table 2). Using the TIMER database, the ALKBH7 expression level was also significantly negatively correlated with 44 of 50 T cell markers in PRAD and THCA and with 31 of 50 T cell markers in PAAD after adjusting for tumor purity (Table 2). These findings further support the hypothesis that ALKBH7 may be involved in inhibiting immune cell infiltration in PAAD, PRAD and THCA.

**TABLE 2:** Correlation analysis between ALKBH7 and gene markers of different types of T cells in TIMER.

Description	Gene markers	THCA				PRAD				PAAD			
		None		Purity		None		Purity		None		Purity	
		Cor	P	Cor	P	Cor	P	Cor	P	Cor	P	Cor	P
Th1	TBX21	−0.199	^a^	−0.192	^a^	−0.2	^a^	−0.17	^a^	−0.194	^b^	−0.143	0.062
	STAT4	−0.317	^a^	−0.317	^a^	−0.281	^a^	−0.216	^a^	−0.207	^b^	−0.179	^c^
	STAT1	−0.451	^a^	−0.436	^a^	−0.421	^a^	−0.339	^a^	−0.355	^a^	−0.302	^a^
	IFNG	−0.231	^a^	−0.221	^a^	−0.206	^a^	−0.144	^b^	−0.277	^a^	−0.238	^b^
	TNF	−0.236	^a^	−0.229	^a^	−0.261	^a^	−0.168	^a^	−0.125	0.096	−0.076	0.32
	IL12A	−0.064	0.152	−0.057	0.206	−0.21	^a^	−0.155	^b^	−0.127	0.09	−0.108	0.159
	IL12B	−0.254	^a^	−0.245	^a^	−0.211	^a^	−0.145	^b^	−0.134	0.075	−0.101	0.187
Th1-like	HAVCR2	−0.395	^a^	−0.382	^a^	−0.364	^a^	−0.3	^a^	−0.352	^a^	−0.28	^a^
	IFNG	−0.231	^a^	−0.221	^a^	−0.206	^a^	−0.144	^b^	−0.277	^a^	−0.238	^b^
	CXCR3	−0.15	^a^	−0.136	^a^	−0.226	^a^	−0.185	^a^	−0.017	0.825	0.046	0.549
	BHLHE40	−0.375	^a^	−0.367	^a^	−0.345	^a^	−0.311	^a^	−0.097	0.196	−0.091	0.238
	CD4	−0.394	^a^	−0.384	^a^	−0.4	^a^	−0.322	^a^	−0.337	^a^	−0.268	^a^
Th2	GATA3	−0.077	0.082	−0.058	0.199	−0.127	^b^	−0.035	0.479	−0.157	^c^	−0.119	0.122
	STAT6	−0.301	^a^	−0.285	^a^	−0.308	^a^	−0.269	^a^	−0.034	0.65	−0.019	0.806
	STAT5A	−0.273	^a^	−0.266	^a^	−0.24	^a^	−0.164	^a^	−0.072	0.339	−0.006	0.942
	IL13	−0.074	0.094	−0.071	0.116	−0.061	0.177	−0.088	0.073	−0.058	0.441	−0.049	0.521
Th17	STAT3	−0.356	^a^	−0.336	^a^	−0.449	^a^	−0.385	^a^	−0.338	^a^	−0.298	^a^
	IL17A	−0.135	^a^	−0.13	^b^	−0.139	^b^	−0.028	0.565	−0.235	^b^	−0.228	^b^
Tfh	BCL6	−0.231	^a^	−0.202	^a^	−0.316	^a^	−0.306	^a^	−0.26	^a^	−0.238	^b^
	IL21	−0.144	^b^	−0.133	^b^	−0.139	^b^	−0.116	^c^	−0.089	0.234	−0.048	0.532
Treg	FOXP3	−0.377	^a^	−0.363	^a^	−0.332	^a^	−0.337	^a^	−0.264	^a^	−0.199	^b^
	CCR8	−0.421	^a^	−0.402	^a^	−0.451	^a^	−0.396	^a^	−0.392	^a^	−0.342	^a^
	STAT5B	−0.209	^a^	−0.189	^a^	−0.465	^a^	−0.409	^a^	−0.126	0.093	−0.127	0.098
	TGFB1	−0.05	0.264	−0.042	0.359	−0.179	^a^	−0.193	^a^	0.07	0.353	0.133	0.083
Resting Treg	FOXP3	−0.377	^a^	−0.363	^a^	−0.332	^a^	−0.337	^a^	−0.264	^a^	−0.199	^b^
	IL2RA	−0.414	^a^	−0.404	^a^	−0.414	^a^	−0.356	^a^	−0.397	^a^	−0.334	^a^
Effector Treg	FOXP3	−0.377	^a^	−0.363	^a^	−0.332	^a^	−0.337	^a^	−0.264	^a^	−0.199	^b^
T-cell	CCR8	−0.421	^a^	−0.402	^a^	−0.451	^a^	−0.396	^a^	−0.392	^a^	−0.342	^a^
	TNFRSF9	−0.38	^a^	−0.361	^a^	−0.454	^a^	−0.377	^a^	−0.362	^a^	−0.309	^a^
Effector	CX3CR1	−0.17	^a^	−0.162	^a^	−0.383	^a^	−0.262	^a^	−0.262	^a^	−0.236	^b^
T-cell	FGFBP2	0.055	0.217	0.051	0.259	−0.118	^b^	−0.094	0.056	−0.22	^b^	−0.196	^c^
	FCGR3A	−0.336	^a^	−0.329	^a^	−0.38	^a^	−0.317	^a^	−0.388	^a^	−0.326	^a^
Naive T-cell	CCR7	−0.293	^a^	−0.276	^a^	−0.274	^a^	−0.217	^a^	−0.178	^c^	−0.124	0.106
	SELL	−0.355	^a^	−0.352	^a^	−0.4	^a^	−0.331	^a^	−0.249	^a^	−0.183	^c^
Effector memory	DUSP4	−0.29	^a^	−0.278	^a^	−0.162	^a^	−0.153	^a^	−0.111	0.137	0.09	0.242
T-cell	GZMK	−0.253	^a^	−0.242	^a^	−0.258	^a^	−0.19	^a^	−0.216	^b^	−0.149	0.052
	GZMA	−0.242	^a^	−0.235	^a^	−0.228	^a^	−0.151	^b^	−0.222	^b^	−0.173	^c^
Resident memory	CD69	−0.324	^a^	−0.311	^a^	−0.389	^a^	−0.306	^a^	−0.335	^a^	−0.297	^a^
T-cell	CXCR6	−0.282	^a^	−0.269	^a^	−0.292	^a^	−0.188	^a^	−0.339	^a^	−0.283	^a^
	MYADM	−0.144	^a^	−0.123	^b^	−0.335	^a^	−0.306	^a^	−0.186	^c^	−0.159	^c^
General memory	CCR7	−0.293	^a^	−0.276	^a^	−0.274	^a^	−0.217	^a^	−0.178	^c^	−0.124	0.106
T-cell	SELL	−0.355	^a^	−0.352	^a^	−0.4	^a^	−0.331	^a^	−0.249	^a^	−0.183	^c^
	IL7R	−0.415	^a^	−0.4	^a^	−0.458	^a^	−0.394	^a^	−0.425	^a^	−0.38	^a^
Exhausted T cell	PDCD1	−0.152	^a^	−0.154	^a^	−0.088	^c^	−0.072	0.142	−0.117	0.119	−0.044	0.568
	CTLA4	−0.317	^a^	−0.304	^a^	−0.151	^a^	−0.115	^c^	−0.227	^b^	−0.159	^c^
	LAG3	−0.211	^a^	−0.207	^a^	−0.031	0.486	0.001	0.98	−0.074	0.323	−0.047	0.543
	HAVCR2	−0.395	^a^	−0.382	^a^	−0.364	^a^	−0.3	^a^	−0.352	^a^	−0.28	^a^
	GZMB	−0.231	^a^	−0.23	^a^	−0.162	^a^	−0.114	^c^	−0.33	^a^	−0.273	^a^
	CXCL13	−0.264	^a^	−0.262	^a^	−0.192	^a^	−0.147	^b^	−0.218	^b^	−0.161	^c^
	LAYN	−0.073	0.098	−0.074	0.101	−0.237	^a^	−0.189	^a^	−0.217	^b^	−0.162	^c^

c *p* < 0.05.

b *p* < 0.01.

a *p* < 0.001.

### The ALKBH7 Coexpression Network Relevant Signalling Pathways

**FIGURE 12:**
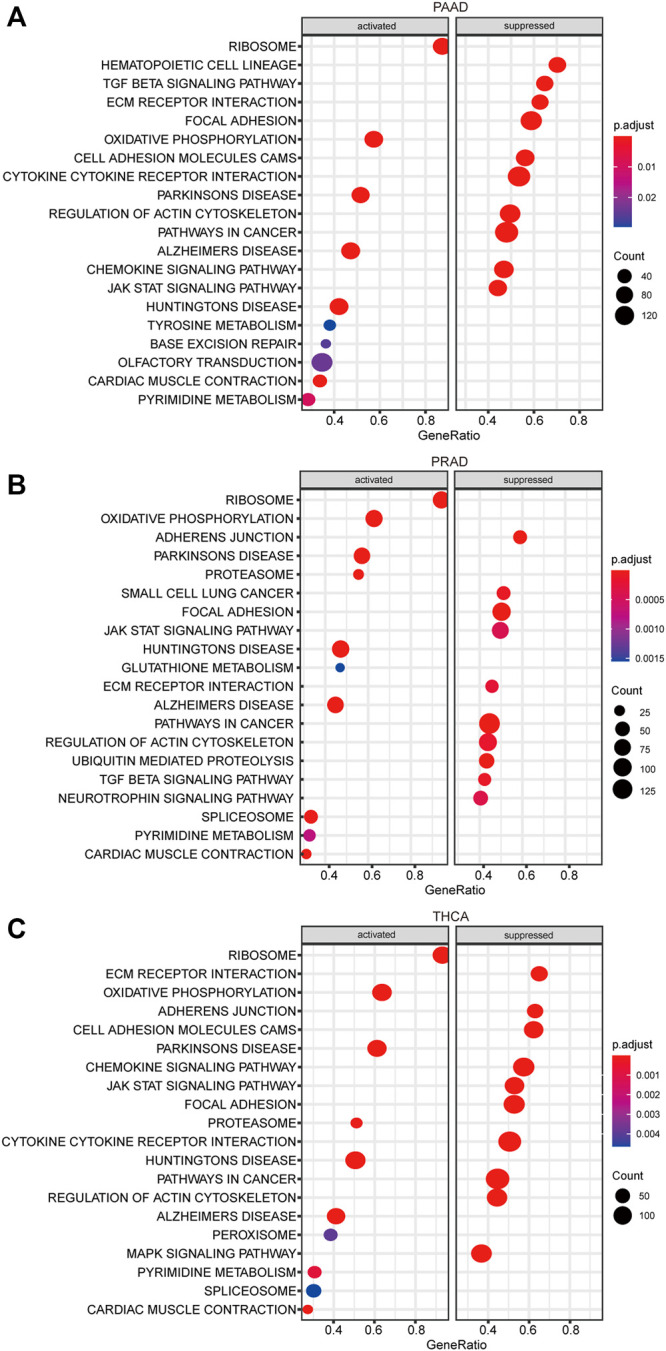
KEGG enrichment analysis of ALKBH7. **(A)** Top 20 enriched KEGG pathways in PAAD. **(B)** Top 20 enriched KEGG pathways in PRAD. **(C)** Top 20 enriched KEGG pathways in THCA.

The aforementioned results identified significant associations between ALKBH7 expression and the prognosis and immunity of cancers. Considering the robust correlation between ALKBH7 expression and PAAD, PRAD and THCA, GSEA was performed to investigate the potential signalling pathways of ALKBH7 in these cancers. The results presented in Figure 12 indicate that genes coexpressed with ALKBH7 are enriched in the regulation of immune and inflammatory responses and are negatively associated with these pathways, such as the JAK/STAT signalling pathway and TGF-β signalling pathway. These results suggested that ALKBH7 expression might play an essential role in human cancers by suppressing the immune response of the TME.

## Discussion

ALKBH7 is a mitochondrial protein involved in programmed necrosis, fatty acid metabolism and obesity development. In this paper, a comprehensive pan-cancer study of ALKBH7 revealed the potential prognostic and immunotherapeutic value of ALKBH7 in human cancers. First, significantly higher ALKBH7 expression was detected in most cancers compared to paired normal tissues, consistent with previous studies. Cai et al. observed high ALKBH7 expression in ovarian plasmacytoma (Cai et al., 2021), and Peng et al. found high ALKBH7 expression in hepatocellular carcinoma (Peng et al., 2021). However, ALKBH7 expression correlates with clinical parameters (age, sex and pathological stage) in only a few patients with cancer. For example, in BLCA, ALKBH7 expression correlated with the pathological stage of the tumor. Interestingly, ALKBH7 expression has some prognostic value for some cancers. For example, in a univariate survival analysis, ALKBH7 expression was significantly associated with four clinical survival datasets (OS, DSS, DFI and PFI) in patients with PAAD; in Kaplan–Meier survival estimates, downregulated ALKBH7 expression was significantly associated with shorter OS and DSS of patients with UCEC. These results suggest that ALKBH7 is a potential prognostic biomarker.

Next, ALKBH7 expression in different immune subtypes and molecular subtypes of human cancers was explored to determine its potential mechanism of action. ALKBH7 expression was significantly different in different immune subtypes and molecular subtypes in many cancer types, suggesting that ALKBH7 is a promising diagnostic pan-cancer biomarker and participates in immune regulation. Moreover, we documented significant differences in ALKBH7 expression in different immune and molecular subtypes of BRCA, PRAD and UCEC. In fact, differential ALKBH7 expression was detected in the cancers listed above and their normal tissue, indicating that ALKBH7 might play a role in the growth and progression of cancers.

Tumor cells use the immune checkpoint pathway to suppress immune cells and achieve immune escape (Topalian et al., 2015). Based on this principle, immune checkpoint inhibitors (ICIs) have emerged as new therapeutic approaches for cancer treatment (Muenst et al., 2016) and have been successfully applied in the clinic. The most commonly used ICI predictive biomarkers are programmed cell death ligand-1 (PD-L1), microsatellite instability (MSI) and tumor mutational burden (TMB) (Wang Y. et al., 2021). In addition, a study by Malta et al. found that a high tumor stemness index was associated with reduced PD-L1 expression in most cancers (Malta et al., 2018). In the present study, immunotherapy biomarkers (TMB and MSI) and the tumor stemness index showed significant associations with ALKBH7 in some cancers. Moreover, a strong relationship between the expression of ALKBH7 and ICP genes was identified. These results indicate that ALKBH7 has a strong association with ICIs.

Based on accumulating evidence, the tumor microenvironment (TME) is involved in tumor progression and significantly affects the treatment response and clinical outcome (Wu and Dai, 2017; Hinshaw and Shevde, 2019). Tumor-infiltrating lymphocytes (TILs) in the TME have been proven to be an independent predictor of the prognosis of patients with cancer and immunotherapeutic efficacy (Azimi et al., 2012). Our study found that ALKBH7 was related to the immune, stromal, and ESTIMATE scores and immune cell infiltration in the TME of most human cancer types, especially in PAAD, PRAD and THCA. Then, we explored the function of ALKBH7 in PAAD, PRAD and THCA by performing a KEGG analysis. ALKBH7 and its coexpression network were indeed involved in the regulation of the immune response and inflammatory response. In summary, these results strongly indicated the potential of ALKBH7 as a target of anticancer immunotherapy.

Overall, our pan-cancer analysis of ALKBH7 is the first to explore the relationship between ALKBH7 expression in human cancers and clinical prognostic factors, immune subtypes, molecular subtypes, immune checkpoints (ICPs), tumor mutational burden (TMB), microsatellite instability (MSI), tumor stemness index, tumor microenvironment (TME) and tumor-infiltrating lymphocytes (TILs). This information contributes to the understanding of the function of ALKBH7 in cancer development and its role in immunology. However, more experimental studies are required to explore the specific mechanisms of ALKBH7 action in cancer.

## Data Availability

The datasets presented in this study can be found in online repositories. The names of the repositories and accession numbers can be found in the article/Supplementary Material.
