# Supplementary material for: A Pan-Cancer Analysis Reveals the Prognostic and Immunotherapeutic Value of ALKBH7
Source: Front Genet. 2022 Feb 11;13:822261. doi: 10.3389/fgene.2022.822261 (PMC8873580; doi:10.3389/fgene.2022.822261)
Supplement: Supplementary file 1 [file Table1.PDF]

Supplementary Table 1. Correlation analysis of ALKBH7 expression with immune infiltration level in TIMER.

Cancer	Purity		B Cell		CD8+ T Cel		CD4+ T Cel		Macrophage		Neutrophil		Dendritic Cell	
	Cor	Pvalue	Cor	Pvalue	Cor	Pvalue	Cor	Pvalue	Cor	Pvalue	Cor	Pvalue	Cor	Pvalue
ACC	0.2160908	0.0644362	-0.0171967	0.8851822	-0.1081275	0.3625219	0.1717699	0.1461968	0.0165749	0.8893067	0.1192042	0.3151426	0.1195923	0.3135584
BLCA	0.1765986	0.0006552	0.1333413	0.0109875	-0.2553652	7.38E-07	-0.0593673	0.2579211	-0.0282369	0.5907688	-0.2670777	2.40E-07	-0.2835478	3.55E-08
BRCA	-0.0433192	0.1721379	-0.1526125	1.65E-06	-0.3011423	6.58E-22	-0.0035012	0.9136375	-0.1626518	2.94E-07	-0.2167315	1.43E-11	-0.1517209	2.60E-06
CESC	0.0930838	0.1215289	0.3014079	3.17E-07	-0.0470398	0.4380338	0.1470274	0.0143141	0.2416708	4.82E-05	-0.1865579	0.0018188	-0.1533845	0.0107178
CHOL	0.1909114	0.2647048	-0.3114115	0.0686059	-0.1864023	0.2836513	-0.1433984	0.4111871	-0.2050211	0.2374151	-0.330393	0.0525657	-0.2095124	0.2270708
COAD	0.1675883	0.0006873	-0.0801655	0.1076374	-0.3369688	3.09E-12	-0.1171825	0.0187589	-0.2136987	1.48E-05	-0.2102505	2.19E-05	-0.16915	0.0006605
DLBC	0.1114579	0.4822272	-0.1423727	0.5730565	0.0259449	0.9111147	-0.159234	0.4905472	0.232531	0.3104241	-0.5716334	0.0067855	-0.3671536	0.1015783
ESCA	0.1887939	0.0109185	-0.0170917	0.8203567	-0.2088265	0.0049041	0.2072811	0.0053675	0.0048077	0.9489281	-0.1570244	0.0352823	0.0961341	0.1992246
GBM	0.0929593	0.2781693	0.22696	0.0105983	0.2049281	0.0193431	-0.1772724	0.0420053	0.0371518	0.6735421	-0.1048098	0.235328	-0.2945536	0.0006072
HNSC	0.1953826	1.25E-05	0.3463984	7.26E-15	0.2404206	1.17E-07	0.0922556	0.0433565	0.2610512	5.76E-09	-0.0430147	0.347527	0.0938332	0.0394697
KICH	0.2991569	0.0146836	-0.0667087	0.5975162	-0.1370156	0.2764344	-0.007484	0.9528185	-0.0861013	0.4952579	-0.2342216	0.0603908	-0.2079659	0.0964383
KIRC	0.084901	0.0682688	-0.1589334	0.000632	-0.2157872	5.19E-06	-0.172646	0.0001986	-0.2509483	7.32E-08	-0.3155659	4.77E-12	-0.1825337	9.01E-05
KIRP	0.0984616	0.1139312	-0.1083515	0.0835879	-0.1756428	0.004661	0.2481177	5.60E-05	-0.0352311	0.5800504	-0.018353	0.7692267	-0.0325704	0.6039601
LGG	0.0696106	0.1281675	-0.2032581	7.50E-06	-0.4278934	1.06E-22	0.0451613	0.3255018	-0.0845655	0.0661207	-0.1629037	0.000364	-0.0877617	0.0556979
LIHC	0.2345691	1.04E-05	-0.0424793	0.4322418	-0.0399279	0.4617388	-0.1699408	0.0015588	-0.1854283	0.0005786	-0.1380181	0.0102712	-0.0466304	0.3913772
LUAD	0.0295733	0.5119675	0.0639411	0.1601686	-0.2037899	5.81E-06	0.0519363	0.2541139	-0.0134725	0.7672694	-0.1352625	0.0028948	-0.0453801	0.3171056
LUSC	0.145759	0.0013959	0.1787312	9.45E-05	-0.0594689	0.1957258	-0.0765334	0.0957022	-0.1267434	0.0055706	-0.1778285	9.59E-05	-0.0412846	0.3698052
MESO	0.1207005	0.2682922	-0.3071943	0.004478	-0.3611219	0.0007387	0.1053652	0.3401459	-0.3180858	0.003192	-0.0080658	0.9419508	-0.3072438	0.0044712
OV	0.0315826	0.4881876	0.0502961	0.271439	0.1390654	0.0022605	0.0027233	0.9525464	0.0237079	0.6043663	0.0687554	0.1325277	0.0845093	0.0643152
PAAD	0.2204465	0.0036622	-0.1920138	0.0118713	-0.4059273	3.62E-08	0.2419076	0.0015315	-0.2813218	0.0001935	-0.2841173	0.0001659	-0.3556486	1.81E-06
PCPG	0.1326007	0.0866308	0.2150828	0.0052489	-0.1782021	0.0212196	0.1899898	0.0139243	0.0349774	0.6545979	-0.1723595	0.0259244	0.2381448	0.0019411
PRAD	0.1402099	0.0041209	-0.2929925	1.34E-09	-0.4274871	6.54E-20	-0.158134	0.0012982	-0.27368	1.40E-08	-0.3173943	3.84E-11	-0.3855251	3.73E-16
READ	0.1530287	0.0710632	-0.0454507	0.595217	-0.3712868	6.80E-06	-0.1497614	0.0784664	-0.2440487	0.0037889	-0.2960958	0.0004217	-0.0980263	0.2509505
SARC	0.0163663	0.7988155	-0.1355782	0.0361993	-0.2445229	0.0001299	0.1308716	0.0432467	0.0196981	0.7638834	-0.1108577	0.0852652	-0.0720985	0.2648925
SKCM	0.1541893	0.000931	-0.0835429	0.0773256	-0.2469546	1.65E-07	0.1102432	0.0198716	-0.096879	0.0392929	-0.2661552	9.09E-09	-0.0930442	0.0495625
STAD	0.0506029	0.3252073	-0.0189492	0.7167542	0.17408	0.0007713	0.0152747	0.7708688	-0.0477093	0.3601269	0.0246033	0.6366662	0.0649594	0.2119204
TGCT	-0.0996862	0.2280269	0.1768007	0.0321773	0.0474485	0.5682077	0.0045453	0.9565773	0.0684894	0.4097868	-0.208004	0.0114682	0.1032454	0.2149336
THCA	0.0297039	0.5122643	-0.3552708	8.74E-16	-0.0900138	0.0471047	-0.2434822	5.12E-08	-0.3521492	1.08E-15	-0.3432012	6.18E-15	-0.408718	5.93E-21
THYM	-0.1052358	0.2608963	-0.0790408	0.4031924	0.1266348	0.1794014	0.2801037	0.002906	0.167694	0.0745211	-0.2263527	0.0154494	0.1889352	0.044092
UCEC	-0.0652511	0.2647454	-0.0142341	0.8095996	0.0635579	0.2815294	-0.1253685	0.0325279	0.0006939	0.9905801	-0.170806	0.0033591	0.0092112	0.8754587
UCS	0.0250569	0.8572708	0.1756203	0.2084457	0.2932263	0.0330972	-0.0487906	0.7286383	0.0214012	0.879104	0.1101456	0.432369	0.3073202	0.0251917
UVM	-0.0165694	0.8855128	-0.0914839	0.4350345	-0.1705546	0.1380689	0.0645802	0.5794111	0.1161815	0.3605935	-0.0466378	0.6871173	0.0829157	0.48247

*p<0.05, **p<0.01, ***P<0.001
