# Supplementary material for: A Pan-Cancer Analysis Reveals the Prognostic and Immunotherapeutic Value of ALKBH7
Source: Front Genet. 2022 Feb 11;13:822261. doi: 10.3389/fgene.2022.822261 (PMC8873580; doi:10.3389/fgene.2022.822261)
Supplement: Supplementary file 3 [file Table2.PDF]

Supplementary Table 2. The correlation between ALKBH7 expression and the ESTIMATE score in human cancers.

CancerType	StromalScore		ImmuneScore		ESTIMATEScore	
	Cor	Pvalue	Cor	Pvalue	Cor	Pvalue
ACC	-0.065457644	0.565811212	-0.122005842	0.283473923	-0.108057449	0.342449389
BLCA	-0.164103424	0.000851668	-0.166159941	0.000731005	-0.172440113	0.000453537
BRCA	-0.085762276	0.004360814	0.002192775	0.941975217	-0.038396641	0.202342619
CESC	0.020571535	0.719879499	0.022233809	0.698309483	0.021730813	0.704811836
CHOL	-0.224453024	0.187521185	-0.111196911	0.517088656	-0.137451737	0.42257646
COAD	-0.213050204	3.26E-06	-0.016403284	0.722439606	-0.123641759	0.007251502
DLBC	-0.279852366	0.054375923	-0.292118975	0.04432277	-0.385692575	0.007123124
ESCA	-0.020600908	0.794498808	0.010972944	0.889668976	-0.000935699	0.990574979
GBM	-0.067882831	0.381579268	0.060955857	0.432120168	0.004957962	0.949100685
HNSC	-0.067323852	0.131940631	0.076245488	0.087906165	0.017475081	0.696002953
KICH	-0.351311189	0.004307585	-0.226223776	0.070112828	-0.282561189	0.022888964
KIRC	-0.268503983	3.24E-10	-0.072928542	0.091956121	-0.173192318	5.79E-05
KIRP	-0.114221155	0.05245606	0.084495685	0.151846665	0.00605586	0.918308715
LAML	-0.248581387	0.002143293	-0.162558383	0.046219322	-0.200857442	0.013521206
LGG	-0.085128418	0.050384369	0.069282636	0.111446345	0.013591131	0.755060891
LIHC	-0.160303365	0.001893004	-0.107755989	0.037293663	-0.138412697	0.007386293
LUAD	-0.04184876	0.337986223	0.075313866	0.084411626	0.01530762	0.726057006
LUSC	-0.139535558	0.001758848	0.032064413	0.473804168	-0.046591216	0.297851791
MESO	-0.292947781	0.006368026	-0.023406764	0.830323744	-0.164036039	0.131091585
OV	-0.046910151	0.362274286	0.022829501	0.65760056	-0.017665977	0.731624108
PAAD	-0.470104657	4.62E-11	-0.312760857	2.35E-05	-0.408793643	2.00E-08
PCPG	-0.112752892	0.128508262	0.062969461	0.396735845	-0.018660954	0.801841594
PRAD	-0.262078857	3.20E-09	-0.153485187	0.000588759	-0.219570418	7.80E-07
READ	-0.157785977	0.041790626	0.082188463	0.290684503	-0.052647717	0.498833931
SARC	-0.013680693	0.825122108	0.088660309	0.151542434	0.054805888	0.375790583
SKCM	-0.138070015	0.002694619	-0.055735011	0.227219858	-0.095919938	0.03746923
STAD	0.004528843	0.930315202	0.114368415	0.026834078	0.06806144	0.188377612
TGCT	-0.29553824	0.000191738	0.098285154	0.221951955	-0.079072561	0.326116952
THCA	-0.265974607	1.22E-09	-0.181328919	3.92E-05	-0.230727214	1.49E-07
THYM	-0.231206381	0.011557739	0.293248825	0.001263165	0.118622703	0.198536427
UCEC	-0.056536621	0.186266884	0.096832127	0.02342706	0.03833814	0.370269786
UCS	-0.29391661	0.028270544	0.220710868	0.102102113	0.003075871	0.982189804
UVM	-0.164252227	0.145196241	-0.234927332	0.036163275	-0.217182372	0.053142694

*p<0.05, **p<0.01, ***P<0.001
