# Supplementary material for: A Pan-Cancer Analysis Reveals the Prognostic and Immunotherapeutic Value of ALKBH7
Source: Front Genet. 2022 Feb 11;13:822261. doi: 10.3389/fgene.2022.822261 (PMC8873580; doi:10.3389/fgene.2022.822261)
Supplement: Supplementary file 4 [file Presentation2.PDF]

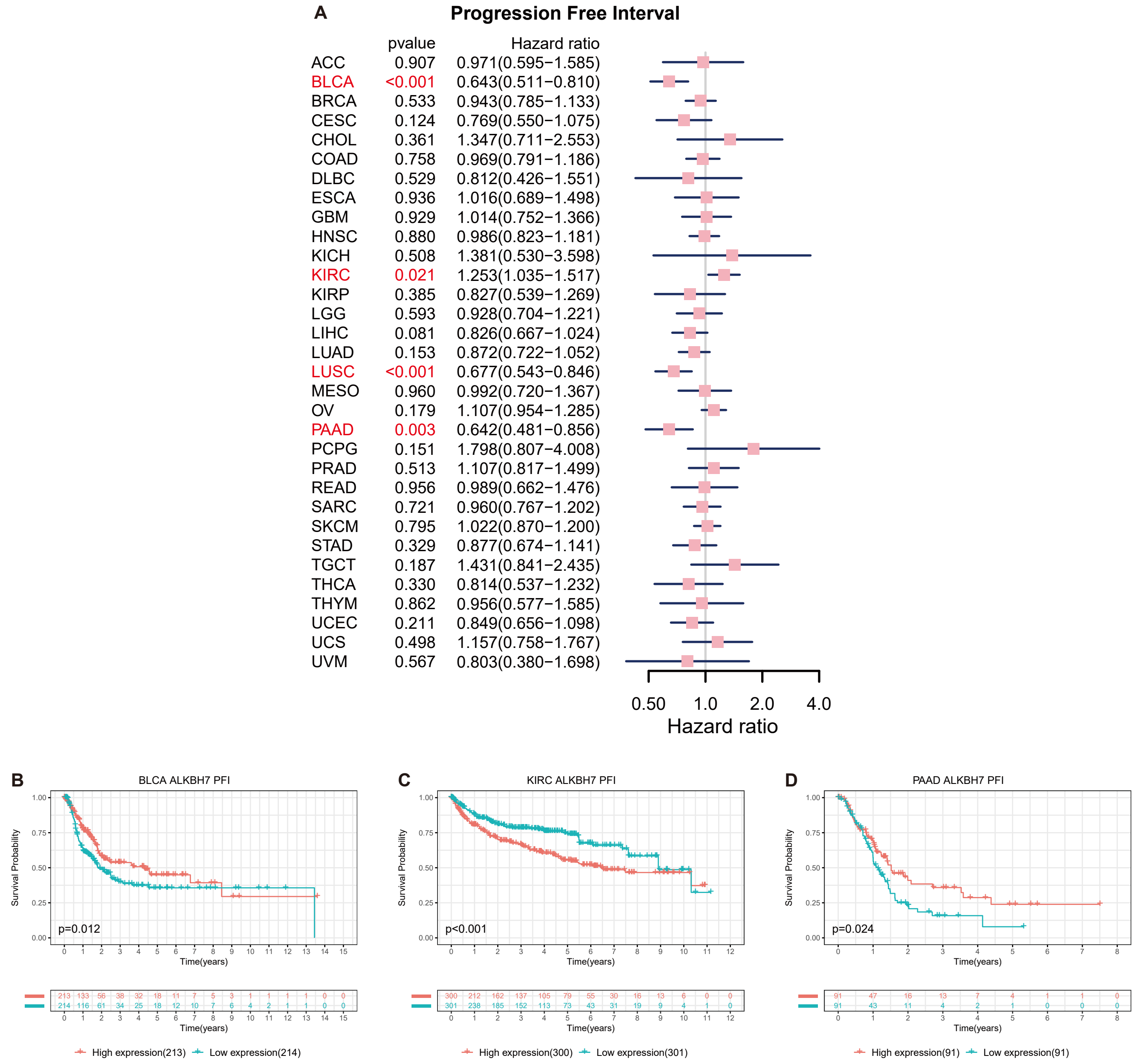

Supplementary Figure 2. Associations between ALKBH7 expression and PFI in cancer patients. **(A)** Hazard ratio forest plot of ALKBH7 in 32 cancers; Kaplan-Meier survival curves of PFI for patients stratified according to different expression profiles of ALKBH7 in BLCA **(B)**, KIRC **(C)** and PAAD **(D)**.
